# The survey dataset of The Influence of theory of planned behaviour on purchase behaviour on social media

**DOI:** 10.1016/j.dib.2022.108239

**Published:** 2022-05-03

**Authors:** Ying Zhou, Alexa Min-Wei Loi, Garry Wei-Han Tan, Pei-San Lo, WeiLee Lim

**Affiliations:** a UCSI Graduate Business School, UCSI University, No. 1 Jalan Menara Gading, UCSI Heights, Cheras, Wilayah Persekutuan Kuala Lumpur 56000, Malaysia; b Faculty of Business and Management, UCSI University Sarawak Campus, Lot 2976, Block 7, Muara Tebas Land District, Sejingkat, Kuching, Sarawak 93450, Malaysia; c Faculty of Business and Management, UCSI University, No. 1 Jalan Menara Gading, UCSI Heights, Cheras, Wilayah Persekutuan Kuala Lumpur 56000, Malaysia

**Keywords:** Theory of planned behaviour, Social shopping, Social commerce, User attitudes, Social norms, Perceived behavioural control, Malaysia

## Abstract

The research aims to study the correlations between attitudes of users, social norms, perceived behavioural control, and purchase behaviours. The research population consists of social media users in Malaysia. The data was collected from 205 respondents via a self-administered online survey. The theory of planned behaviour acts as the underlying theory in the research. Next, descriptive and hypothesis-testing quantitative analysis were adopted to probe the relationships between the variables. Moreover, G*Power was used to identify the minimum sample size, and SPSS v.22 was employed to examine the datasets.

**Specification Table**

**Table:** 

Subject	Marketing
Specific subject area	Purchase Behaviour in Social Commerce
Type of data	TableRaw Data (.xls)Survey QuestionnaireDescriptive Statistics
How data were acquired	Online surveys. A copy of the questionnaire is uploaded on Mendeley Repository Name: MendeleyDirect URL to data: https://data.mendeley.com/datasets/nzy5528nst/2
Data format	RawanalysedFiltered
Parameter for data collection	The respondents must possess at least one social media account to be eligible to partake in the research.
Description of data collection	The online survey questionnaires were distributed conveniently to Internet users located in Malaysia.
Data source location	Malaysia
Data accessibility	Dataset is uploaded on MendeleyRepository Name: MendeleyDirect URL to data: https://data.mendeley.com/datasets/nzy5528nst/2

**Value of the Data**

• The data provide insights on the factors which influence the purchase behaviour of social media users in Malaysia.
• The insights can provide suggestions to enhance the virtual and extended interaction between retailers and customers in the digitalised market.
• External literature can reuse the data to probe the purchasing behaviour of different generations under the impact of social media influence.
• The data can be used by academician to illustrate a course in statistical exploitation of survey data that focuses on structural equations modelling.
• The research model can be adapted with moderation or moderating effect for researchers to explore the purchase behaviour of the younger generation in the respective countries.

## 1 Data Description

**Fig. 1:**
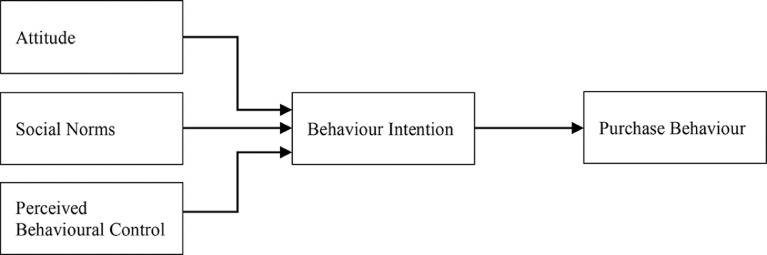
Research model.

The raw data file and the survey questionnaire employed are provided alongside the data article as supplementary documents. The research model is built on the theory of planned behaviour shown in Fig. 1, a social cognitive model used in social psychology to explain the complexity of human behaviour where the behaviour intention is the direct antecedent used to forecast real conduct of a person willingness to put effort to conduct the behaviour concerned [1,3].

The empirical data are illustrated with 6 tables. Table 1 summarizes the constructs adopted from past research, with respective definitions and sources. Table 2 presents the demographic profile of the sample, categorized by gender, age, ethnicity, income level, and occupation. Table 2 also includes the behavioural background of participants, in terms of their experiences with social media. Table 3 displays the mean, standard deviation, and reliability of the measured constructs. The reliability of the data is probed using Cronbach's alpha, as shown in Table 3. The Cronbach's alpha score is above the minimum threshold of 0.7 (ATTD = 0.922; SN = 0.919; PBC = 0.934; PB = 0.912), hence the data is reliable and consistent [7,^8^,^14^,^21^,22]. Table 4 shows the model summary of the coefficient of determination (R^2^) model. The *R*-value (0.918) and R-square (0.842) in Table 4 demonstrated that the effectiveness of the model in determining the dependant variables is substantial, as based on the recommended explained variance (R^2^) values by Hair et al. [5], R^2^ of 0.75, 0.50, and 0.25 signify substantial, moderate, and weak predictive power [9,^15^,^17^,19]. Table 5 shows the readings of ANOVA. Finally, the model coefficients, which include the Beta and standard error of the measured constructs are presented in Table 6. The findings from Tables 5 and 6 indicate significant relationships between all the constructs, by which attitudes of users, social norms, and perceived behaviour control are positively related to the purchase behaviour of social media users in Malaysia; and can be used to predict the purchase behaviour of social media users in Malaysia.

**Table 1:** Constructs, definitions, and sources.

Constructs	Definitions	Measurement Items	Sources
Attitude (ATTD)	Attitude is a person's positive or negative feeling that is influenced by the convictions required for action.	ATTD1: Advertisement on social media can help me to be aware of the existence of product. ATTD2: Advertisement on social media more easily attract my attention when compared to other advertising channels. ATTD3: Prominent keywords such as promotion and discount on social media will attract my attention to seek more production information. ATTD4: I ever purchase a product that I have become aware of through social media.	[1,4]
Social Norms (SN)	Social norms are the perceived social pressures of public moral beliefs that are expected of important reference objects to engage in an action.	SN1: My family influence my purchasing decision towards social media marketing. SN2: People around me think that I should purchase products through social media. SN3: I feel good if many people purchased products through social media. SN4: My friends encourage me to purchase products through social media.	[1,4]
Perceived behavioural Control (PBC)	This is an individual resources and opportunities must, to some extent, determine the possibility of behavioural achievement.	PBC1: Frequency product advertisement on social media led me to buy. PBC2: I will choose social media as a reference channel for purchasing in the future. PBC3: I will recommend my friend choose social media as a reference channel for making purchase intention in the future. PBC4: I will recommend family choose social media as a reference channel for making purchase intention in the future.	[1,4]
Purchase Behaviour (PB)	This refers an individual willingness to conduct an action to buy product or services for consumption.	PB1: I am willing to buy a product promoted on social media. PB2: There is a high probability that I would purchase a product because of the impact of social media. PB3: I am easily influenced by advertisement on social media and further make a purchase behaviour. PB4: I had the experience of buying a product due to the influence of social media.	[1,4]

**Table 2:** Demographic of participants (*N* *=* 205)*.*

Demographic variables	Category	Frequency	Percentage
Gender	Male	93	45.4
	Female	112	54.6
Age	17–22	40	19.5
	23–28	64	31.2
	29–34	52	25.4
	35–40	39	19.0
	Others	10	4.9
Ethnic	Malay	21	10.2
	Chinese	167	81.5
	Indian	16	7.8
	Others	1	0.5
Occupation	Student	49	23.9
	Businessman	46	22.4
	Housemaker	16	7.8
	Employee	92	44.9
	Retired	2	1.0
Annual income	Less than RM30,000	36	17.6
	RM 30,001–RM 50,000	46	22.4
	RM 50,001–RM 70,000	47	22.9
	RM 70,001–RM 90,000	26	12.7
	RM 90,001 and above	50	24.4
Social media usage	Yes	205	100.0
	No	0	0.0
Experience of purchasing under influence of social media	Yes	188	91.7
	No	17	8.3
Experience of purchasing under the influence of reviews and ratings	Yes	171	83.4
	No	34	16.6
Average time spent on the Internet per week	1–4 h	55	26.8
	5–10 h	51	24.9
	10–20 h	29	14.1
	20–40 h	42	20.5
	40 h and above	28	13.7
Attention to advertisements on social media	Yes	132	64.4
	No	73	35.6

**Table 3:** Mean, standard deviation and reliability of measured constructs.

	Attitude (ATTD)	Social Norms (SN)	Perceived behavioural Control (PBC)	Purchase Behaviour (PB)
Mean	3.330	3.085	3.128	3.261
Standard Deviation	1.088	1.149	1.181	1.051
Cronbach's alpha	0.922	0.919	0.934	0.912

**Table 4:** Coefficient of determination (R^2^) model.

Model	R	R Square	Adjusted R Square	Std. Error of the Estimate
1	.918^a^	.842	.840	.42086

a Predictors: (Constant), PBC = Perceived Behavioural Control; ATTD = Attitude; SN = Social Norms.

**Table 5:** ANOVA^a^.

Model		Sum of Squares	df	Mean Square		F	Sig.
1	Regression	189.936	3		63.312	347.451	.000^b^
	Residual	35.602	201		.177		
	Total	225.538	204				

a Dependent Variable: PB = Purchase Behaviour.

b Predictors: (Constant), BC = Perceived Behavioural Control; ATTD = Attitude; SN = Social Norms.

**Table 6:** Coefficients^a^.

		Unstandardized Coefficients		Standardized Coefficients			
Model		B	Std. Error		Beta	*t*	Sig.
1	(Constant)	.369	.095			3.873	.000
	ATTD	.515	.057		.533	9.021	.000
	SN	.126	.068		.138	1.861	.064
	SPBC	.252	.072		.283	3.487	.001

a Dependent Variable: PB **=** Purchase Behaviour.

## 2 Experimental Design, Materials and Methods

The research employed quantitative methods with the deployment of self-administered online survey questionnaires as the measurement tool [16,^17^,^18^,20]. Moreover, the survey questionnaires consist of 16 measurement items for the 4 latent constructs probed. The measurement items were adopted and adapted from past literature [4,11] and anchored on a 5-point Likert scale which ranged from 1: “Strongly Disagree” to 5: “Strongly Agree”. 340 questionnaires were distributed with a non-probability, convenience sampling method via Google Forms to the individual who uses social media, howbeit only 219 filled-pout responses were collected. Snowball sampling was used where the survey link is shared via WeChat Moments for voluntary participations. No incentives were given for the survey. Of the 219 empirical data collected, only 205 responses were qualified and eligible for analysis about data filtering, the remaining respondents were eliminated due to inexperience with social media. The filtered dataset still exceeds the minimum sample size determined using G*Power 3 with a statistical power of 0.8, margin error of 0.05, and effect size of 0.15, with 3 predictors was used to determine the minimum sample size, as recommended by past literature [10,^16^,18]. Besides, in reference to Roscoe et al. [12] rule of thumb, a sample size between 30 and 500 should be sufficient for most studies [6,13]. SPSS v.22 was employed to conduct the data analysis, particularly the Cronbach's alpha to examine the reliability and validity [2], then structure equation modelling to estimate the coefficients of the latent variable, which is believed to be the appropriate estimation methods for multiple regression analysis [8,^23^,24].

## Ethical Statements

The self-administered survey that is non-experimental in nature was conducted under complete anonymity for the participants. No personal or sensitive information that can be used to identify the respondents were collected. Besides, the consent of the respondents to partake in the online survey were seek before the survey was executed by including an electronic informed consent in the online survey form. Furthermore, the data redistribution policies of the social media platforms were complied with.

## CRediT authorship contribution statement

**Ying Zhou:** Conceptualization, Methodology, Formal analysis, Writing – original draft, Writing – review & editing. **Alexa Min-Wei Loi:** Conceptualization, Methodology, Formal analysis, Writing – original draft, Writing – review & editing. **Garry Wei-Han Tan:** Conceptualization, Methodology, Formal analysis, Writing – original draft, Writing – review & editing. **Pei-San Lo:** Conceptualization, Methodology, Formal analysis, Writing – original draft, Writing – review & editing. **WeiLee Lim:** Conceptualization, Methodology, Formal analysis, Writing – original draft, Writing – review & editing.

## Declaration of Competing Interest

The authors declare no conflict of interest.

## Data Availability

Survey dataset of The Influence of theory of planned behaviour on purchase behaviour on social media (Original data) (Mendeley Data).

## Appendix Supplementary materials

Image, application 1

Image, application 2
