# Supplementary material for: The survey dataset of The Influence of theory of planned behaviour on purchase behaviour on social media
Source: Data Brief. 2022 May 3;42:108239. doi: 10.1016/j.dib.2022.108239 (PMC9111892; doi:10.1016/j.dib.2022.108239)
Supplement: Supplementary file 2 [file mmc2.docx]

**Social Media Marketing on Consumer’s Purchasing Behaviour**

**Survey Questionnaire**

| The purpose of this survey is to explore social media users’ opinions and perceptions towards social media marketing on purchasing behaviour. Please answer all questions to the best of your knowledge. There are no wrong responses to any of these statements. All responses are completely confidential.  Your participation in this survey is entirely voluntary and anonymous. All the information provided will be kept private and confidential. You have the right to withdraw from the survey at any time. To withdraw your participation from this survey, simply close your browser. By completing this survey questionnaire, you are indicating that you are willing to take part in this research study and granted consent. (*for minors below 18 years old, you are required to seek your parent's consent before proceeding with the survey*)  To protect your privacy, this survey will not ask you to provide personally-identifying information. If you have questions at any time about this study, please feel free to contact the researcher Zhou Ying at 431435508@qq.com  Thank you for your participation. |
| --- |

**Instructions:**

1. There are **two** (2) sections in this questionnaire. Please answer **ALL** questions in ALL sections.
2. Completion of this form will take you approximately 5 to 10 minutes.
3. The contents of this questionnaire will be kept **strictly confidential**.

Section A: Demographic Profile

*In this section, we are interested in your background in brief. Please tick your answer and your answers will be kept strictly confidential.*

QA1: Gender: ❑ Male ❑ Female

QA2: Age: ❑ 17 - 22 Years Old ❑ 23 - 28 Years Old ❑ 29 - 34 Years Old

❑ 35 - 40 Years Old ❑ Others, please specify ________

QA3: Ethnic: ❑ Malay ❑ Chinese ❑ Indian

❑ Others, please specify ________

QA4: Occupation:

❑ Student ❑ Businessman ❑ Homemaker

❑ Employee ❑ Retired

QA5: Annual income range:

❑ Less than RM30,000 ❑ RM30,001-RM50,000 ❑ RM50,001-RM70,000

❑ RM70,001-RM90,000 ❑ More than RM90,001

QA6: Do you use social media?

❑ Yes ❑ No

QA7: Do you have experience purchasing because of the influence of social media?

❑ Yes ❑ No

QA8: Do reviews and ratings on social media affect your purchasing decision?

❑ Yes ❑ No

QA5: On average, how many hours do you spend on the internet per week?

❑ 1 hour – 4 hours ❑ 5 hours – 10 hours ❑ 10 hours – 20 hours

❑ 20 hours – 40 hours ❑ More than 40 hours

QA7: Do you pay attention to the advertisement on social media?

❑ Yes ❑ No

Section B: Factors that influence your intention to purchase and to making purchase decision

*This section is seeking your opinion regarding social media marketing influences on your purchasing intention. Social media marketing refers to the use of social media platform in connecting with the customer to building brand reputation, increase sales, and drive traffics on retailer’s website. The major social media platform are Facebook, Instagram, Twitter, LinkedIn, Pinterest, YouTube, and Snapchat.*

*Respondents are asked to indicate the extent to which they agreed or disagreed with each statement using 5 Likert scale* [(1) = strongly disagree; (2) = disagree; (3) = neutral; (4) = agree; (5) = strongly agree] *response framework. Please circle one number per line to indicate the extent to which you agree or disagree with the following statements.*

| **No** | **Questions** | **Strongly Disagree** | **Disagree** | **Neutral** | **Agree** | **Strongly Agree** |
| --- | --- | --- | --- | --- | --- | --- |
| B1 | Purchase Behaviour (PB) |  |  |  |  |  |
| PB1 | I am willing to buy a product promoted on social media. | 1 | 2 | 3 | 4 | 5 |
| PB2 | There is a high probability that I would purchase a product because of the impact of social media. | 1 | 2 | 3 | 4 | 5 |
| PB3 | I am easily influenced by advertisements on social media and further make a purchase behaviour. | 1 | 2 | 3 | 4 | 5 |
| PB4 | I had the experience of buying a product due to the influence of social media. | 1 | 2 | 3 | 4 | 5 |

| **No** | **Questions** | **Strongly Disagree** | **Disagree** | **Neutral** | **Agree** | **Strongly Agree** |
| --- | --- | --- | --- | --- | --- | --- |
| B2 | Attitude (ATTD) |  |  |  |  |  |
| ATTD 1 | Advertisement on social media can help me to be aware of the existence of products. | 1 | 2 | 3 | 4 | 5 |
| ATTD 2 | Advertisements on social media more easily attract my attention when compared to other advertising channels. | 1 | 2 | 3 | 4 | 5 |
| ATTD 3 | Prominent keywords such as promotion and discount on social media will attract my attention to seek more production information. | 1 | 2 | 3 | 4 | 5 |
| ATTD 4 | I ever purchase a product that I have become aware of through social media. | 1 | 2 | 3 | 4 | 5 |

| **No** | **Questions** | **Strongly Disagree** | **Disagree** | **Neutral** | **Agree** | **Strongly Agree** |
| --- | --- | --- | --- | --- | --- | --- |
| B3 | Social Norms (SN) |  |  |  |  |  |
| SN1 | My family influence my purchasing decision towards social media marketing. | 1 | 2 | 3 | 4 | 5 |
| SN2 | People around me think that I should purchase products through social media. | 1 | 2 | 3 | 4 | 5 |
| SN3 | I feel good if many people purchased products through social media. | 1 | 2 | 3 | 4 | 5 |
| SN4 | My friends encourage me to purchase products through social media. | 1 | 2 | 3 | 4 | 5 |

| **No** | **Questions** | **Strongly Disagree** | **Disagree** | **Neutral** | **Agree** | **Strongly Agree** |
| --- | --- | --- | --- | --- | --- | --- |
| B4 | Perceived Behavioural Control (PBC) |  |  |  |  |  |
| PBC1 | Frequency product advertisement on social media led me to buy. | 1 | 2 | 3 | 4 | 5 |
| PBC2 | I will choose social media as a reference channel for purchasing in the future. | 1 | 2 | 3 | 4 | 5 |
| PBC3 | I will recommend my friend choose social media as a reference channel for making purchase intention in the future. | 1 | 2 | 3 | 4 | 5 |
| PBC4 | I will recommend family choose social media as a reference channel for making purchase intention in the future. | 1 | 2 | 3 | 4 | 5 |

*Thank you for your time and cooperation.*

*~ The End ~*
